# Muscarinic Modulation of High Frequency Oscillations in Pedunculopontine Neurons

**DOI:** 10.3389/fneur.2013.00176

**Published:** 2013-11-06

**Authors:** Nebojsa Kezunovic, James Hyde, Belen Goitia, Veronica Bisagno, Francisco J. Urbano, Edgar Garcia-Rill

**Affiliations:** ^1^Department of Neurobiology and Developmental Sciences, Center for Translational Neuroscience, University of Arkansas for Medical Sciences, Little Rock, AR, USA; ^2^IFIBYNE-CONICET-UBA, University of Buenos Aires, Buenos Aires, Argentina

**Keywords:** calcium channels, carbachol, gamma oscillations, G-proteins, muscarinic receptors, 5′-[β-thio] diphosphate trilithium salt, 5′-[γ-thio] triphosphate trilithium salt

## Abstract

We previously reported that persistent application of the non-specific cholinergic agonist carbachol (CAR) increased the frequency of calcium channel-mediated oscillatory activity in pedunculopontine nucleus (PPN) neurons, which we identified as dependent on voltage-gated, high-threshold P/Q-type channels. Here, we tested the hypothesis that M2 muscarinic receptors and G-proteins associated with M2 receptors mediate the increase in oscillatory frequency in PPN neurons. We found, using depolarizing ramps, that patch clamped 9–12 day old rat PPN neurons (*n* = 189) reached their peak oscillatory activity around −20 mV membrane potential. Acute (short duration) application of CAR blocked the oscillatory activity through M2 muscarinic receptors, an effect blocked by atropine. However, persistent (long duration) application of CAR significantly increased the frequency of oscillatory activity in PPN neurons through M2 receptors [40 ± 1 Hz (with CAR) vs. 23 ± 1 Hz (without CAR); *p* < 0.001]. We then tested the effects of the G-protein antagonist guanosine 5′-[β-thio] diphosphate trilithium salt (GDP-β-S), and the G-protein agonist 5′-[γ-thio] triphosphate trilithium salt (GTP-γ-S). We found, using a three-step protocol in voltage-clamp mode, that the increase in the frequency of oscillations induced by M2 cholinergic receptors was linked to a voltage-dependent G-protein mechanism. In summary, these results suggest that persistent cholinergic input creates a permissive activation state in the PPN that allows high frequency P/Q-type calcium channel-mediated gamma oscillations to occur.

## Introduction

The pedunculopontine nucleus (PPN) is part of the reticular activating system (RAS), which modulates the fast oscillating rhythms between thalamus and cortex manifested in the EEG during the activated states of waking and rapid eye movement (REM) sleep (1). The PPN is located ventral to the inferior colliculus and partially embedded in the lateral portion of the superior cerebellar peduncle. We found that all PPN cell types maximally fire at gamma frequency (2), and exhibit intrinsic oscillatory beta/gamma band frequency (20–29 Hz/30–80 Hz, respectively) activity dependent on high threshold, voltage-dependent P/Q-type calcium channels, which can be modulated by carbachol [CAR; Ref. (3)]. The present study was undertaken to determine the mechanism behind the modulation of high frequency oscillatory activity of individual PPN neurons by CAR.

The PPN receives cholinergic input from the contralateral PPN and laterodorsal tegmental nuclei (4). Muscarinic receptors (mAChR) have five subtypes M1–M5. M2 and M4 receptors are known to be G_i_-protein coupled. These receptors increase the membrane conductance to potassium ions while reducing calcium currents, which hyperpolarizes the membrane potential (5). M1, M3, and M5 receptors are thought to be excitatory, and are linked to G_q_ proteins. Activation of M1 receptors will depolarize the membrane potential by blocking potassium currents (6), probably through the intracellular calcium signaling pathway mediated by inositol 1,4,5-triphosphate. Previous studies showed that PPN cholinergic neurons were predominantly hyperpolarized by the activation of post-synaptic M2 receptors (7, 8). Different *in situ* hybridization studies confirmed that cholinergic cells in the PPN primarily express M2 receptors, although low levels of M3 and M4 mRNA have been detected (9, 10). Previous studies from our laboratory showed that 73% of PPN output neurons were inhibited through M2 receptors, 13% were excited through M1 and nicotinic receptors, and 7% showed biphasic responses (11).

G-protein inhibition of both N- and P/Q-type calcium channels by CAR has been extensively characterized in previous studies (12–16). Both voltage-dependent and voltage-independent G-protein pathways that inhibit calcium channels have been described in a number of systems (17). Voltage-dependent modulation is initiated by G-protein activation and mediated by the G_βγ_ subunit (14, 15). When the membrane potential is driven to positive voltages, this shift can be partially reversed. On the contrary, other G-protein mediated pathways block calcium channels by mechanisms insensitive to membrane potential (i.e., voltage-independent), and have been described as pertussis toxin-insensitive G-proteins (14, 16, 17).

Although the dependence of PPN oscillations on P/Q-type calcium channels has been established (3), modulation of this activity by cholinergic input and its G-protein-dependent mechanism is poorly understood. In this study, we found that direct and voltage-independent modulation of P/Q-type calcium channels by G-protein mediated intracellular pathways underlies the effects of CAR on gamma frequency activity in the PPN.

## Materials and Methods

### Slice preparation

Rat pups aged 9–12 days from adult timed-pregnant Sprague-Dawley rats (280–350 g) were anesthetized with ketamine (70 mg/kg, i.m.) until the tail pinch reflex was absent. This age range was selected due to the developmental decrease in REM sleep in the rat that occurs between 10 and 30 days (18). In addition, it is well known that most of the changes in PPN transmitter interactions occur across the 12–16 day period [reviewed in Ref. (19)]. This window of investigation enabled sampling from a baseline period before the epoch of the greatest transitions that peak at 12–16 days and continues until ∼20 days, as determined by our previous body of work on the PPN (19). However, we did not observe either age-related (9–12 days) or cell type related changes in the properties described below, in agreement with our previous work (3). Pups were decapitated and the brain was rapidly removed and cooled in oxygenated sucrose-artificial cerebrospinal fluid (sucrose-aCSF). The sucrose-aCSF consisted of (in millimolar): 233.7 sucrose, 26 NaHCO_3_, 3 KCl, 8 MgCl_2_, 0.5 CaCl_2_, 20 glucose, 0.4 ascorbic acid, and 2 sodium pyruvate. Sagittal sections (400 μm) containing the PPN were cut and slices were allowed to equilibrate in normal aCSF at room temperature for 1 h before recording. The aCSF was composed of (in millimolar): 117 NaCl, 4.7 KCl, 1.2 MgCl_2_, 2.5 CaCl_2_, 1.2 NaH_2_PO_4_, 24.9 NaHCO_3_, and 11.5 glucose. Slices were recorded at 37°C while perfused (1.5 ml/min) with oxygenated aCSF (95% O_2−_5% CO_2_) in an immersion chamber containing the sodium channel blocker tetrodotoxin citrate (TTX, 3 μM), and the following neurotransmitter receptor antagonists: the selective NMDA receptor antagonist 2-amino-5-phosphonovaleric acid (APV, 40 μM), the competitive AMPA/kainate receptor antagonist 6-cyano-7-nitroquinoxaline-2,3-dione (CNQX, 10 μM), the glycine receptor antagonist strychnine (STR, 10 μM), and the specific GABA_A_ receptor antagonist gabazine (GBZ, 10 μM), all purchased from Sigma-Aldrich (St. Louis, MO, USA). Our experimental protocols were approved by the Institutional Animal Care and Use Committee of the University of Arkansas for Medical Sciences, and were in agreement with the National Institutes of Health guidelines for the care and use of laboratory animals.

### Whole-cell patch clamp recordings

Differential interference contrast optics was used to visualize neurons using an upright microscope (Nikon FN-1, Nikon, Japan). Whole-cell recordings were performed using borosilicate capillary glass tubing pulled on a P-1000 puller (Sutter Instrument Company, Novato, CA, USA). Electrodes were filled with high-K^+^ intracellular solution (in millimolar): 130 K^+^-methanesulfonate, 10 NaCl, 10 Phosphocreatine, 10 HEPES, 1 EGTA, 4 Mg-ATP, 0.4 Na_2_GTP, 2 MgCl_2_, 10 sucrose. To study calcium currents, electrodes were filled with high-Cs^+^/QX-314 intracellular solution (in millimolar): 110 Cs^+^-methanesulfonate, 40 HEPES, 1 EGTA, 5 QX-314, 10 TEA-Cl, 4 Mg-ATP, 0.4 Na_2_GTP, 10 Phosphocreatine, 2 MgCl_2_. Cesium is a widely used potassium channel blocker, while QX-314 (Sigma-Aldrich, St. Louis, MO, USA) blocks sodium channels intracellularly. Osmolarity was adjusted to ∼270–290 mOsm/L and pH to 7.3. The pipette resistance was 2–4 MΩ. All recordings were made using a Multiclamp 700B amplifier (Molecular Devices, Sunnyvale, CA, USA). Data was recorded in both current and voltage-clamp modes. Analog signals were low-pass filtered at 2 kHz, and digitized at 5 kHz using a Digidata-1440A interface and pClamp10 software (Molecular Devices). The recording region was located mainly in the *pars compacta* in the posterior PPN, immediately dorsal to the superior cerebellar peduncle. This area of the PPN has been shown to have the highest density of cells (11, 20). Gigaseal and further access to the intracellular neuronal compartment was achieved in voltage-clamp mode, a series of hyperpolarizing steps run to identify cell type, followed by setting the holding potential at −50 mV [which is close to the average resting membrane potential of PPN neurons and also inactivates T-type calcium channels, (3)]. Soon after rupturing the membrane, the intracellular solution reached equilibrium with the pipette solution without significant changes in either series resistance or membrane capacitance values. The configuration was then changed to current-clamp mode, another series of steps were run to confirm cell type, and the membrane potential was then depolarized using either 1 s (3) or 30 s depolarizing current ramp protocols, allowing the assessment of longer periods of high frequency oscillatory activity in PPN neurons. Previous studies have shown that there are three major cell types in the PPN which manifest (a) low threshold spikes (LTS) upon release from hyperpolarization (type I), which are non-cholinergic, (b) Ia current that slows the return from release from hyperpolarization (type II), two-thirds of which are cholinergic, and (c) both Ia and LTS, one third of which are cholinergic [reviewed in Ref. (1, 19)]. All the cells analyzed in this research were type I, II, or III. Calcium channel-mediated oscillatory activity in PPN neurons was studied in current-clamp mode, in the presence of synaptic blockers (see above) and TTX. Previous studies showed that all PPN cell types manifest calcium channel-mediated gamma band activity, and that the only difference is that type II cells showed lower amplitude gamma oscillations than type I and III PPN cells (3). Calcium currents were studied using the high-Cs^+^/QX-314 pipette solution also in the presence of synaptic blockers and TTX at a holding potential of −50 mV. Time course of activation (τ_ON_) and deactivation (τ_OFF_) of calcium currents were obtained after fitting individual currents to the function *y* = *y*_0_ + *a* × exp (−Time (ms)/τ_(ON or OFF)_). No significant rundown due to intracellular dialysis of PPN neuronal activity was observed (up to 30 min) using these methods. Membrane voltage was depolarized from the −50 mV holding potential after compensating series resistance (after compensation, final *R*s values ranged 5–10 MΩ; >12 kHz bandwidth). Three-pulse square voltage steps (consisting of a test pulse to 0 mV, or pre-pulse, for 20 ms followed by a strong depolarizing step to +50 mV for 20 ms, a brief return to the holding potential of −50 mV for 10 ms, followed by another test pulse to 0 mV, or post-pulse for 20 ms, durations designed to allow full recovery of high threshold, voltage-dependent calcium currents) were used to study G-protein modulation of PPN calcium currents by CAR (12–16). This protocol allowed us to characterize whether G-protein inhibition of calcium currents was mediated by a voltage-dependent (i.e., when CAR inhibition was less potent during the post-pulse than during the pre-pulse), or by a voltage-independent pathway (i.e., when a similar inhibition by CAR was observed during both pre- and post-pulses). After determining cell type, we tested the control responses to depolarizing ramps, namely the induction of gamma band oscillations. The effects of acute application of CAR (30 μM) then were recorded over the first 10 min of perfusion, during which ramps were applied every minute. On the other hand, the persistent effect of CAR was quantified in an extracellular solution containing the synaptic blockers, nicotinic antagonists, and CAR (30 μM) applied continuously for 20 min or longer before current ramps were applied to elicit oscillations.

### Drug application

Bath-applied drugs were administered to the slice via a peristaltic pump (Cole-Parmer, Vernon Hills, IL, USA) and a three-way valve system such that drugs reached the slice in 1.5 min. The sodium channel blocker tetrodotoxin citrate (TTX, 3 μM) was purchased from Sigma-Aldrich (St. Louis, MO, USA), as well as cholinergic agonist carbachol (CAR, 30 μM). Cholinergic antagonists were also purchased from Sigma, mecamylamine (MEC, 10 μM, a nicotinic receptor antagonist), methoctramine (MTO, 2 μM, a M2 muscarinic receptor antagonist), pirenzepine (PIR, 10 μM, a M1 muscarinic receptor antagonist), and atropine (ATR, 10 μM, a non-specific muscarinic receptor antagonist) to confirm the receptor type responsible for the changes in oscillatory frequency in PPN neurons. The calcium channel blockers were purchased from Alomone labs (Alomone.com). We used ω-conotoxin-GVIA (ω-CgTX, 2.5 μM, applied >20 min), a specific N-type calcium channel blocker, to confirm the effects of CAR on P/Q-type calcium channels. Both ω-CgTX and ω-agatoxin-IVA (ω-AgA, 100–200 nM, applied >20 min) were used to confirm that both beta/gamma oscillations and calcium currents recorded from PPN neurons were P/Q- and N-type calcium channel mediated (3). The G-protein antagonist guanosine 5′-[β-thio] diphosphate trilithium salt (GDP-β-S, 1 mM), and the G-protein agonist guanosine 5′-[γ-thio] triphosphate trilithium salt (GTP-γ-S, 0.4 mM) were purchased from Sigma-Aldrich (St. Louis, MO, USA). GDP-β-S and GTP-γ-S were administered intracellularly through the recording micropipette.

### Data analysis

Off-line analyses were performed using Clampfit software (Molecular Devices, Sunnyvale, CA, USA). Comparisons between groups were carried out using either Student’s *t*-test or one-way ANOVA, with Bonferroni correction of *post hoc* testing for multiple comparisons. A repeated-measures ANOVA model was fit for each response using SAS Proc Mixed software (SAS Institute Inc., Cary, NC, USA), and the Bonferroni or Dunn’s *post hoc* tests were further calculated. *t*-Values and degrees of freedom were reported for all linear regression ANOVAs. Differences were considered significant at values of *p* ≤ 0.05. All results are presented as mean ± SEM.

## Results

**Figure 1:**
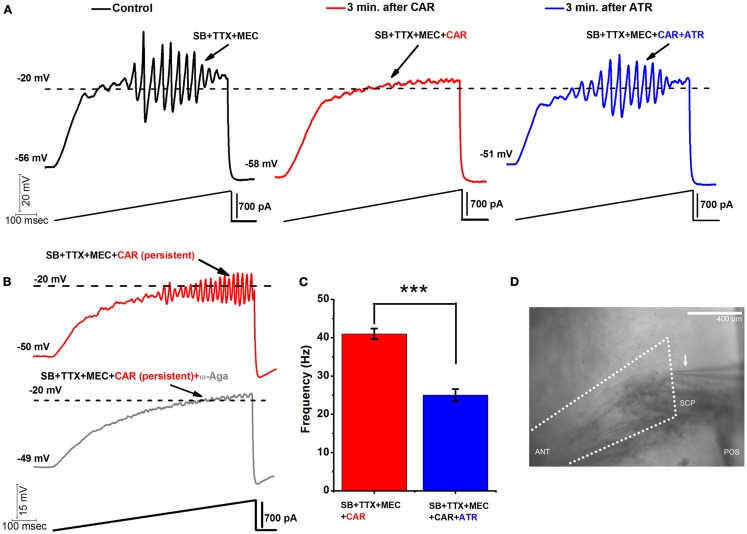
**Acute vs. persistent effect of CAR on the oscillatory behavior of PPN neurons**. **(A)** Representative 1 s long current ramp-induced oscillations of a PPN neuron in SB + TTX + MEC extracellular solution (left record, black). After 3 min of CAR in the extracellular solution, the oscillatory activity diminished (middle record, red). However, the acute effect of CAR on oscillations was reversed by adding ATR to the solution (after 3 min of perfusion with a solution containing CAR + ATR) (right record, blue). **(B)** Representative 1 s long current ramp recording of a PPN neuron in the presence of SB + TTX + MEC + CAR (red record, top). Note that this cell was recorded after persistent exposure to CAR ( >20 min of exposure). Below is the record of the same neuron showing that oscillations were blocked after adding ω-Aga (200 nM) to the extracellular solution (*n* = 5; gray record, bottom). These recordings confirm that P/Q-type calcium channel-mediate oscillatory activity in PPN neurons was induced by persistent activation by CAR. **(C)** Bar graph showing the mean frequency of oscillatory activity in the presence of SB + TTX, the nicotinic receptor blocker MEC, persistent exposure to CAR (red column), and in the presence of SB + TTX + CAR and non-specific cholinergic antagonist ATR (blue column). Note that the mean oscillatory frequency of PPN neurons was significantly higher during persistent exposure to CAR compared to the condition with ATR in the extracellular solution. **(D)** Wide field photomicrograph of the region of the PPN showing the recording electrode (arrow). Anterior is to the left, posterior to the right, with the fibers of the superior cerebellar peduncle (SCP) visible ventral to the recording pipette within the PPN *pars compacta*, which is located immediately dorsal to the posterior end of the peduncle. This was the region selectively sampled, and every cell identified as to PPN cell type. [In **(C)**: ***p < 0.001; calibration bar in **(D)**: 400 μm; abbreviations: CAR, carbachol; SB, synaptic blockers; TTX, tetrodotoxin; MEC, mecamylamine; ATR, atropine; ω-Aga, agatoxin].

Whole-cell patch clamp recordings were performed from a total of 189 PPN neurons and their responses to depolarizing 1 s and 30 s current ramps showed voltage dependence of their oscillatory behavior as previously described (3). The neurons were localized mainly in the *pars compacta* of the posterior PPN, which is easily identified in sagittal sections of the brainstem (Figure 1D) (2, 3, 11). In this study, we recorded from 32 type I, 92 type II, and 65 type III PPN cells. We previously showed that, regardless of cell type, P/Q-type calcium channel activation mediated oscillatory activity in PPN neurons (3). Moreover, we reported that continuous, long duration application of CAR (30 μM) increased the frequency of these oscillations (3). Previous studies described the proportion of mesopontine cells responding to short duration exposure to cholinergic agents. Over 89% of cells in the PPN and LDT of the guinea pig (8), and 95% of rat LDT neurons (7) were hyperpolarized by CAR. We used low levels of current to compensate CAR-mediated changes in membrane potential in order to maintain *V*_m_ in the −50 mV range, as previously reported (3). After studying both the acute and persistent effects of CAR on oscillatory activity in PPN neurons, we undertook pharmacological characterization of the cholinergic receptors responsible for the increase in frequency. Finally, we ascertained the role of G-proteins in the increase in oscillatory frequency induced by CAR, and their association with high-threshold voltage-gated P/Q-type calcium channels.

One of the main characteristics of PPN oscillations is that the membrane potential has to be gradually depolarized up to ∼−20 mV (using 1 s current ramps) in order to activate high threshold, voltage-dependent P/Q-type calcium channels, and to a lesser extent, N-type calcium channels, that mediate high frequency oscillations. Calcium imaging techniques confirmed that these oscillations are indeed occurring in the dendrites of the PPN neurons (21). Although we cannot determine the actual membrane potential of the dendrites without clamping them, we assume that, due to the accumulated membrane capacitance between the electrode and the dendritic compartments, the real threshold of these oscillations is much closer to physiological values and close to action potential (AP) threshold. Representative membrane potential oscillations were recorded using 1 s current ramps (using >300 pA current amplitudes). PPN oscillation amplitudes peaked at around −20 mV and progressively diminished when the membrane potential was depolarized above −10 mV (Figure 1A). A similar result was observed in 29 PPN neurons. We attempted to diminish the decrease in oscillation amplitude by depolarizing membrane potential in a more gradual fashion. We used longer, 30 s ramps (data not shown), achieving a more gradual depolarization that allowed us to detect oscillatory activity at around −40 mV membrane potential (a similar effect was observed in 25 PPN neurons). In addition, lower current levels (100–300 pA) were needed to reach the −20 mV threshold for the peak of the oscillations. Longer duration ramps, therefore, allowed us to increase the number of points sampled at different membrane potentials manifesting a similar pattern of frequencies in the power spectra. However, 30 s protocols were not practical for pharmacological experiments due to their detrimental effect on the overall health of the neurons when applied for prolonged periods.

### Acute vs. persistent effects of CAR on the oscillatory activity of PPN neurons

Release of the neurotransmitter acetylcholine (ACh) by the RAS indirectly leads to cortical fast EEG activity and arousal (22– 25). ACh plays an important role in communication between nuclei of the RAS, particularly between the PPN and Pf. Both nuclei are known to receive the cholinergic afferents (26, 27). In our previous studies, we showed that both PPN and Pf cells have similar mechanisms involving high threshold, voltage-dependent calcium channel-mediated gamma oscillations that can increase in frequency under persistent application of CAR (30 μM) (3, 28). During membrane potential recordings in PPN neurons (in the presence of synaptic blockers, TTX, and MEC), acutely applied CAR had a blocking effect on high-threshold oscillations within 2–3 min after bath application. Figure 1A is an example of a representative ramp-induced membrane potential oscillation observed in a PPN neuron in the presence of synaptic blockers, TTX, and MEC (left record, black). After only 3 min of acute exposure to CAR, oscillations were eliminated (middle record, red). However, adding atropine (ATR, 10 μM) to the extracellular solution antagonized the effects of CAR on oscillatory activity of the same PPN neuron. ATR reversed the blocking effect of CAR, and oscillations returned to the levels prior to the addition of the CAR (right record, blue; the same results were observed in five PPN neurons). The effects of ATR suggest the presence of a fast and reversible acute muscarinic modulation of PPN oscillatory activity.

We previously reported that ω-Agatoxin-IVA (ω-Aga, a specific P/Q-type calcium channel blocker, 200 nM) blocked the oscillatory properties of PPN neurons (3). Here, we show that P/Q-type calcium channels play a central role in CAR-mediated persistent effects since ω-Aga blocked the increase in frequency of oscillations induced by CAR (*n* = 4). Figure 1B shows a control current ramp recording after persistent (20 min) exposure to CAR (red record), and the blocking effect of ω-Aga (gray record) 10 min later in the same representative PPN neuron.

Moreover, we compared the mean frequency of PPN oscillations under the persistent influence of CAR, *n* = 12 (SB + TTX + MEC, see above) against the mean frequency under the persistent effect of CAR and ATR, *n* = 5 (SB + TTX present in the extracellular solution). Statistical comparison showed that the CAR group without ATR (41 ± 1 Hz, red bar) had significantly higher frequency of oscillation compared to the CAR group with ATR in the extracellular solution (25 ± 2 Hz, blue bar) (One-way ANOVA; *df* = 16, *t* = −7.1, *p* < 0.001). These results suggest that long lasting activation of muscarinic receptors induced higher frequency of oscillations in the PPN.

### Acute application of CAR blocked oscillatory activity in PPN neurons through M2 muscarinic receptors

**Figure 2:**
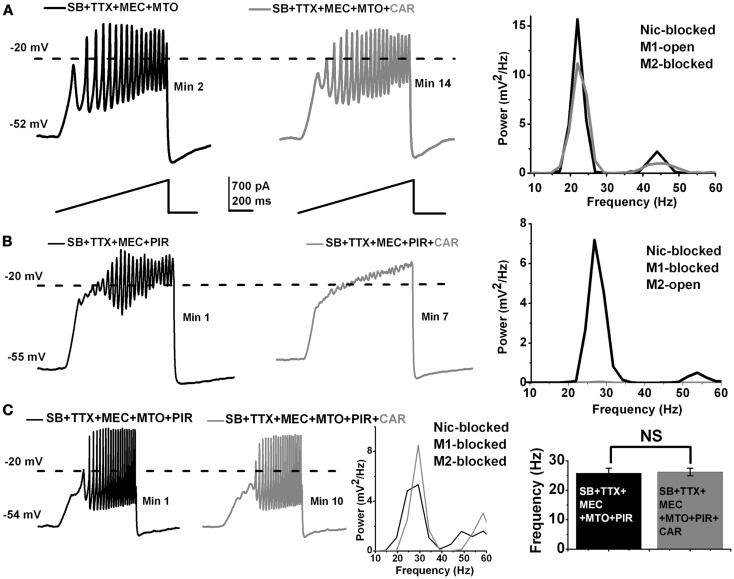
**Acute effect of CAR on M1 and M2 muscarinic receptors in the PPN**. **(A)** Representative membrane potential oscillations recorded during 1 s long ramps in the presence of SB + TTX + MEC, and the M2 muscarinic receptor blocker MTO (left record, black). Adding CAR to the extracellular solution had no effect on the oscillatory behavior of PPN cells, even after 12 min of exposure (right record, gray). The power spectrum of these recordings (right side) shows that there was no shift in the frequency of oscillations after acute exposure to CAR. **(B)** Representative membrane oscillations obtained during 1 s long ramps in the presence of SB + TTX + MEC and M1 muscarinic receptor blocker PIR (left record, black). Adding CAR to the extracellular solution blocked the oscillations after 7 min (right record, gray). The power spectrum of these recordings (right side) shows that acute exposure to CAR completely blocked the oscillations acting through M2 muscarinic receptors. **(C)** Representative membrane oscillations recorded during 1 s long ramps in the presence of SB + TTX and all the cholinergic blockers MEC + MTO + PIR (left record, black). Addition of CAR to the extracellular solution had no effect on the oscillations (left record, gray). The power spectrum of these records shows no shift in frequency after exposure to CAR (middle). The bar graph (right side) shows the mean frequency of oscillations before and after acute exposure to CAR in the presence of SB + TTX + MEC + MTO + PIR. Note that there was no statistical difference in the mean of frequency before (black column, *n* = 4) vs. after (gray column, *n* = 4) addition of CAR to the extracellular solution in the presence of nicotinic and muscarinic blockers. (Abbreviations: CAR, carbachol; SB, synaptic blockers; TTX, tetrodotoxin; MEC, mecamylamine; MTO, methoctramine; PIR, pirenzepine).

We then determined what subtype of muscarinic cholinergic receptor is responsible for the blockade of the oscillatory activity in PPN neurons. Basically, one of the two or both major muscarinic receptors described in the PPN [M1 and M2; Ref. (11)], might be blocking oscillations when activated by CAR. We first tested the role of M1 receptors. To the extracellular solution containing synaptic blockers and TTX, we added MEC (to block nicotinic receptors), and methoctramine (MTO to block M2 muscarinic receptors). We ran ramp protocols to measure the responsiveness of the cells, as well as its steady state oscillatory activity. After 10 min, CAR was added to the extracellular solution, and we recorded 1 s ramps every minute. An example of this protocol appears in Figure 2A (*n* = 7). The recording on the left (black record) taken 2 min after adding MTO shows the oscillations induced, while the recording on the right (red record) shows that 14 min after the addition of MTO and 2 min after the addition of CAR (which would normally block oscillations as shown in Figure 1) had no effect on the induced oscillations. The power spectrum of these recordings (far right side) indicates that there was no shift in the frequency or any blocking effect of the oscillations, even 12 min after CAR exposure (One-way ANOVA; *df* = 13, *t* = 0.17, *p* = 0.87). A small decrease in the power of the oscillations was probably due to a partial rundown of calcium currents, which is to be expected during whole-cell recordings (Figure 2A, right, compare black/MTO vs. gray MTO + CAR line). The results suggest that CAR did not block the oscillations via M1 muscarinic receptor activation.

To determine if M2 muscarinic receptors were in fact responsible for mediating the acute CAR-dependent blocking effect of the oscillations, we used the same extracellular solution as described above except for the addition of pirenzepine (PIR, a M1 muscarinic receptor blocker). By blocking M1 receptors, responses to CAR could be attributed to M2 receptors. Under these conditions, the blocking effect of CAR was immediate (Figure 2B; *n* = 8). The recording on the left (black record) shows induced oscillations 1 min after adding PIR, but the recording on the right shows that the addition of CAR (gray record) eliminated the oscillations within 3 min of adding CAR. The power spectrum of the recordings (far right side) showed a complete blockade of the oscillations after only a few minutes of exposure to CAR (compare black/PIR vs. gray/PIR + CAR line). These results suggest that CAR blocks the oscillations completely through M2 receptors, with no trace of persisting oscillations indicative of an exclusive role of M2 receptors.

In addition, we showed that oscillations were not blocked by CAR when all of the cholinergic blockers (MEC that blocks nicotinic receptors, MTO that blocks M2 muscarinic receptors, and PIR that blocks M1 muscarinic receptors) were present in the extracellular solution (Figure 2C; *n* = 4). The recording on the left (black record) in the presence of MEC + MTO + PIR manifested oscillations, which were not affected by the addition of CAR in the right recording (gray record). The power spectrum from a representative recording in Figure 2C shows no shift in the frequency even 10 min after the cell was exposed to CAR. The graph on the far right side of Figure 2C shows that there was no significant difference in the frequency of oscillations before (26 ± 2 Hz, black bar, *n* = 4) compared to after CAR (26 ± 1 Hz, gray bar, *n* = 4) in an extracellular solution containing all of the cholinergic blockers (One-way ANOVA; *df* = 7, *t* = 0.24, *p* = 0.82). These results suggest that acute activation of M2 muscarinic receptors blocked beta/gamma oscillations in PPN neurons, and that no portion of the oscillations remained that could implicate M1, M3, or M4 receptors in this effect.

### M2 muscarinic receptors mediated the persistent CAR-induced shift in the frequency of PPN oscillations

**Figure 3:**
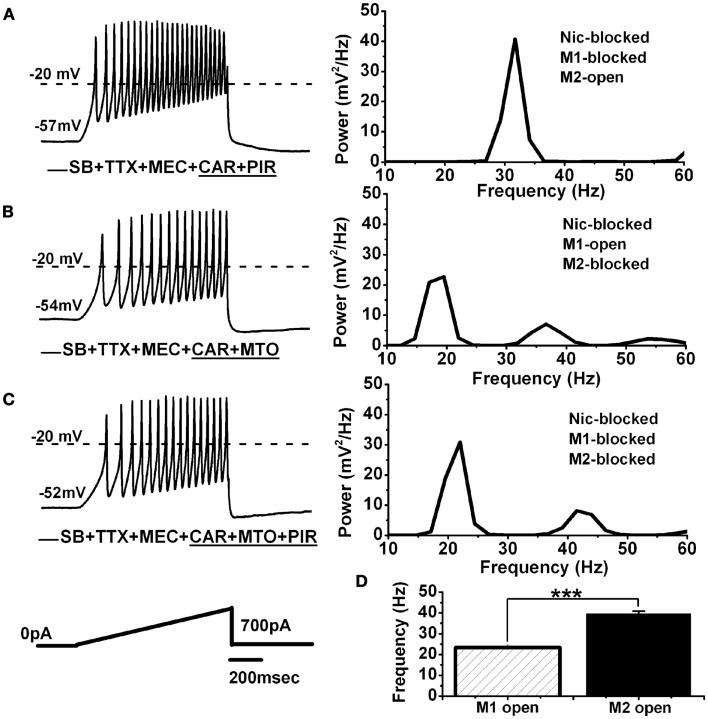
**Persistent effect of CAR on M1 and M2 muscarinic receptors in the PPN**. **(A)** Representative membrane potential oscillations recorded during a 1-s long ramp (black record on the left) in the presence of SB + TTX + MEC + persistent CAR and PIR. Note the higher frequency of oscillations due to M2 muscarinic receptors being activated. The power spectrum of this recording (right side) shows oscillations at gamma frequency. **(B)** Representative membrane potential oscillations recorded during a 1-s long ramp (black record on the left) in the presence of SB + TTX + MEC + CAR and MTO. Note slower oscillatory activity in this cell due to M1 muscarinic receptors being activated and M2 receptors being blocked. The power spectrum of this recording (right side) shows oscillations in the beta frequency range, and reduced gamma band activity. **(C)** Representative membrane oscillations recorded during 1 s long ramp (black record on the left) in the presence of all synaptic blockers including all the cholinergic blockers (MEC + MTO + PIR). The power spectrum on the right side shows oscillatory activity in the beta range, similar to the frequencies observed in the experiment described in **(B)**. **(D)** Bar graph showing the mean frequency of membrane potential oscillations for the cells recorded under persistent exposure to CAR where one group had only M1 receptors activated (striped column, *n* = 7), and the other group had only M2 receptors activated (black column, *n* = 11). Note that both groups of cells were recorded under the same conditions (SB + TTX + MEC), except for the specific M1 vs. M2 receptor blockers (***p < 0.001; abbreviations: CAR, carbachol; SB, synaptic blockers; TTX, tetrodotoxin; MEC, mecamylamine; MTO, methoctramine; PIR, pirenzepine).

We tested the role of M2 receptors using pirenzepine (PIR, 10 μM), after 20 min of CAR application (i.e., CAR-mediated persistent effect). Figure 3A shows an example of membrane oscillations recorded during 1 s long ramps in the presence of CAR + PIR (*n* = 11). The power spectrum on the right shows the frequency of oscillations to be 33 Hz, in the gamma range. In contrast, cells recorded in a similar extracellular condition, except that the M1 receptor blocker PIR was replaced with the M2 receptor blocker MTO, recordings showed reduced gamma band oscillations induced by 1 s long ramps during exposure to CAR + MTO (Figure 3B; *n* = 7). The power spectrum of this record showed a PPN neuron with large amplitude oscillations in the beta range (21 Hz), and almost no gamma band activity (Figure 3B, right). Furthermore, when we added all of the cholinergic blockers mentioned above (MEC, PIR, and MTO) to the extracellular solution with synaptic blockers and TTX (Figure 3C; *n* = 5), the cells oscillated at lower (beta) frequencies, that were similar to those previously reported (3). Statistical analysis showed that PPN cells oscillated significantly faster under persistent application of CAR (Figure 3D) when only M2 receptors were available (MEC + PIR + CAR, 40 ± 1 Hz, black bar, *n* = 11), compared to when only M1 receptors were available (MEC + MTO + CAR, 23 ± 1 Hz, striped bar, *n* = 7) (One-way ANOVA; *df* = 17, *t* = 8.7, *p* < 0.001). These data indicate that persistent activation of M2 muscarinic receptors will trigger the activation of intracellular second messengers that ultimately affect calcium channels responsible for mediating gamma frequency oscillatory activity. We then undertook studies to identify the intracellular mechanisms underlying this phenomenon.

### Effect of CAR on calcium channels in the PPN mediated by a voltage-independent G-protein mechanism

**Figure 4:**
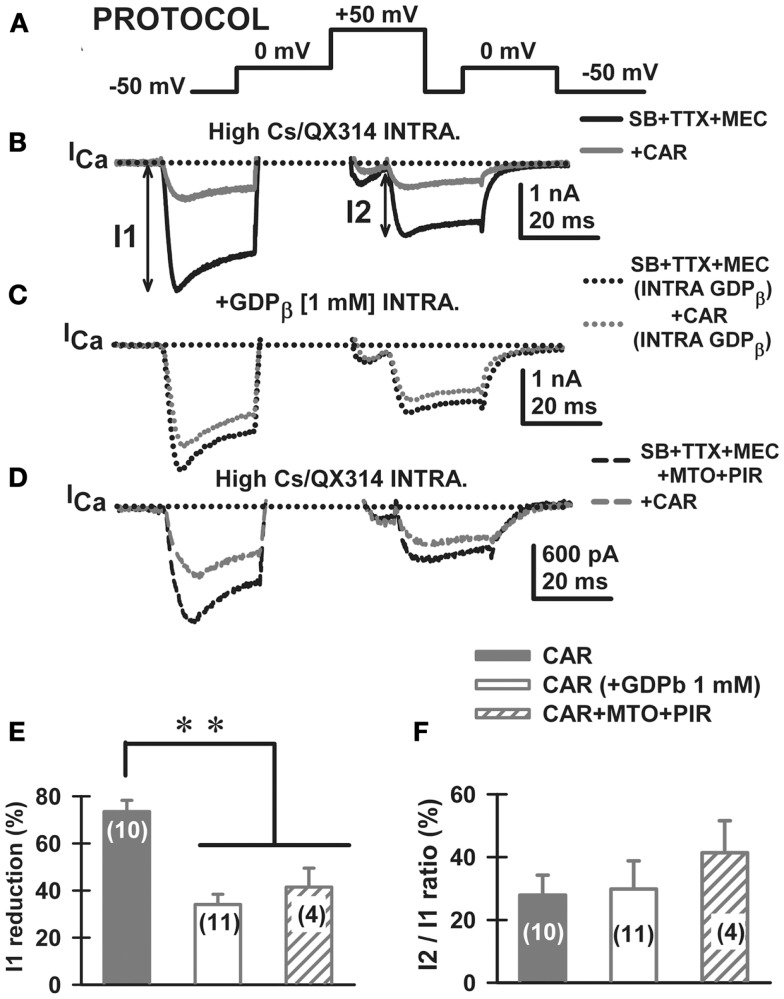
**Carbachol-mediated voltage-dependent G-protein modulation of voltage-gated calcium currents in PPN**. **(A)** Three-pulse protocol (see Materials and Methods) used to study the voltage dependence of G-protein modulation of calcium currents (I_Ca_) in PPN neurons. **(B)** In the presence of synaptic receptor blockers (SB: GBZ + STR + AP5 + CNQX), TTX, and MEC, calcium currents (I_Ca_; black record) were reduced in amplitude when the three-pulse protocol was applied. Indeed, the I_Ca_ observed after the second 0 mV pulse (I2) was always of lower amplitude than the one observed after the first pulse (I1), yielding a I2/I1 ratio <1 in all neurons recorded. Interestingly, CAR (30 μM) reduced the total amount of current during both pulses without affecting calcium current amplitude in I2/I1 ratios (gray record). **(C)** The effects of CAR were prevented in recordings with GDP-β-S in the intracellular solution (1 mM). Note that the black record (before CAR) was similar to the gray record (after CAR). **(D)** The addition of cholinergic receptor antagonists also reduced the response to CAR. **(E)** The effect of CAR (solid column) was significantly reduced when either intracellular GDP-β-S (open column) or extracellular muscarinic receptor antagonists (M2 antagonist methoctramine-MTO + M1 antagonist pirenzepine-PIR) (hatched column) were used. **(F)** Calcium current I2/I1 ratio values (%) were unchanged by CAR under any of the experimental conditions. These results suggest that CAR reduced calcium currents through activation of M2 muscarinic receptors that activate G-proteins. (Abbreviations: CAR, carbachol; SB, synaptic blockers; TTX, tetrodotoxin; MEC, mecamylamine; MTO, methoctramine).

It has been demonstrated that membrane-delimited inhibition of voltage-dependent calcium channels is affected by G_βγ_ subunits of neurotransmitter activated G-proteins (12–16). In particular, CAR has been described by others to reduce calcium currents through a membrane-delimited fast mechanism (17, 29, 30). We employed a three-pulse protocol, previously used in other models, to study G-protein mediated modulation of calcium currents (Figure 4A, PROTOCOL). Using the appropriate conditions to record only calcium currents (3), the protocol consisted of a test pulse to 0 mV (pre-pulse to test the effects on calcium currents when G-proteins are bound) followed by a strong depolarizing step to +50 mV (which at least partially displaces G-protein binding), a brief return to the holding potential of −50 mV, and finally, another test pulse to 0 mV (post-pulse, which tests the effects on calcium currents when G-proteins are at least partially unbound). When a given receptor agonist reduces the amplitude of the pre-pulse more than the post-pulse, a voltage-independent G-protein mechanism underlies such reduction in calcium currents. On the contrary, when a similar inhibition is observed at both test pulses, then a voltage-dependent mechanism can be described. Here, in the presence of the synaptic blockers, TTX, and MEC, the total calcium current (I_Ca_) observed after the post-pulse (I2) was always (*n* = 24 neurons) of lower amplitude than the one observed after the pre-pulse (I1), yielding a I2/I1 ratio <1 in every PPN neuron studied (Figure 4B, black record). Bath application of ω-CgTX + ω-Aga fully eliminated the I_Ca_ observed during three-pulse protocol (*n* = 5 PPN neurons, data not shown), suggesting that the I_Ca_ was generated entirely by P/Q-type calcium channels.

Carbachol (30 μM) reduced the total amplitude elicited by both pulses and showed apparent slower kinetics of the I_Ca_ without affecting calcium current amplitude I2/I1 ratios when compared to control condition (Figure 4B, gray record). The effect of CAR was prevented after adding GDP-β-S to the intracellular pipette solution (see Materials and Methods; Figure 4C). The total blocking effect of CAR (Figure 4E, solid bar) was significantly reduced when either intracellular GDP-β-S (Figure 4E, open bar), or extracellular muscarinic receptor antagonists were used (MEC + MTO + PIR, Figure 4D I_Ca_ before and after CAR; Figure 4E, hatched bar). Calcium current I2/I1 ratio values (%) were unchanged by CAR under all experimental conditions (Figure 4F). These results suggest that CAR reduced calcium currents through activation of M2 muscarinic receptors that activate G-proteins via a voltage-dependent mechanism.

**Figure 5:**
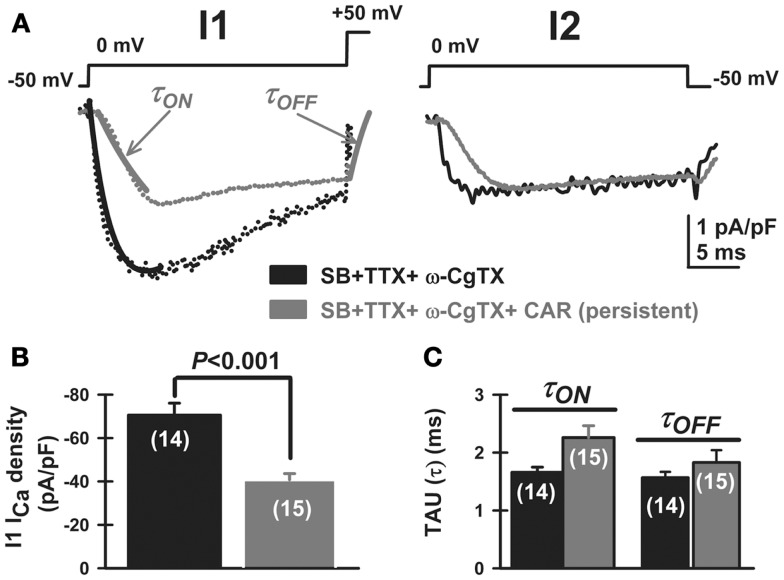
**Carbachol-mediated voltage-dependent G-protein modulation of P/Q-type voltage-gated calcium currents in PPN**. **(A)** Three-pulse protocol used to study the voltage dependence of G-protein modulation of calcium currents (I_Ca_) in PPN neurons after blocking N-type channels with ω-conotoxin-GVIA 2.5 μM. In the presence of synaptic receptor blockers (SB: GBZ + STR + AP5 + CNQX), TTX, MEC, and ω-CgTX-GVIA, calcium current density (I_Ca_; pA/pF, black record) was reduced in amplitude by CAR (gray record) when the three-pulse protocol was applied as described in Figure 4. **(B)** CAR (30 μM) reduced the total amount of current density after the first pulse (before CAR black column, after CAR gray column; Mann–Whitney Rank Sum Test, *p* < 0.001). **(C)** Time course of activation (τ_ON_) and deactivation (τ_OFF_) of calcium currents, obtained after fitting individual currents to the function *y* = *y*_0_ + *a* × exp (−[−Time (ms)/τ_(ON or OFF)_). The effects of CAR on time course were not significantly different (Kruskal–Wallis ANOVA, *H* = 4.33, *p* = 0.23). (Abbreviations: CAR, carbachol; SB, synaptic blockers; TTX, tetrodotoxin; MEC, mecamylamine).

We then studied the effects of CAR on P/Q-type calcium channels, which are the main high-threshold calcium channels involved in PPN oscillatory activity [i.e., after blocking N-type channels with ω-conotoxin-GVIA 2.5 μM; Ref. (3)]. After bath-applying CAR for >20 min, P/Q-type mediated I_Ca_ showed apparently slower activation and deactivation time courses for both the pre-pulse and post-pulse compared to the ω-CgTX-GVIA condition (Figure 5A, τ_ON_ and τ_OFF_ arrows, respectively). In addition, CAR reduced I_Ca_ density (pA/pF) from −71 ± 5 to −41 ± 3 pA/pF (Figure 5B, Mann–Whitney Rank Sum Test, *p* < 0.001). However, mean current density values observed after CAR in the absence or presence of ω-CgTX-GVIA were not significantly different (CAR: 17 ± 5 pA/pF, *n* = 9; CAR + ω-CgTX-GVIA: 29 ± 3 pA/pF; Student’s *t*-test, Kruskal–Wallis ANOVA, *H* = 3.6, *p* = 0.06). No significant differences were observed when comparing τ_ON_/τ_OFF_ values (Figure 5C, Kruskal–Wallis ANOVA, *H* = 4.33, *p* = 0.23). These results suggest that the effects of CAR in reducing oscillations was entirely through P/Q-type calcium channels.

**Figure 6:**
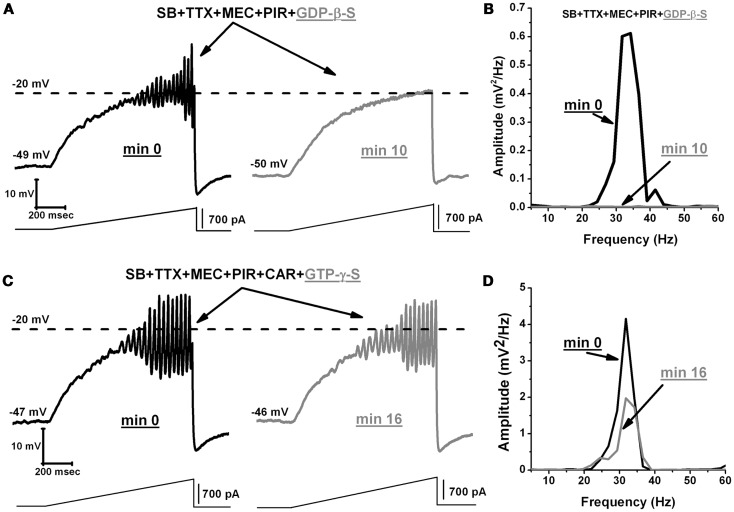
**GDP-β-S blocks oscillatory activity in PPN neurons**. **(A)** Representative membrane potential oscillation recorded during 1 s ramp immediately after rupturing the membrane, in the presence of SB + TTX + MEC + PIR (left record, black). Note the blocking effect of GDP-β-S on the oscillations in the same neuron after 10 min (right record, gray). **(B)** Power spectrum of the records in **(A)** showing elimination of gamma band oscillations by GDP-β-S. **(C)** Representative membrane potential oscillation in the presence of SB + TTX + MEC + PIR + CAR following rupturing the membrane (left record, black). Note that activation of G-proteins by GTP-γ-S did not decrease or further increase the frequency of oscillations (right record, gray). **(D)** Power spectrum of the records in C showing that high frequency oscillation frequency remained similar before (black line) vs. after (gray line) GTP-γ-S. (Abbreviations: CAR, carbachol; SB, synaptic blockers; TTX, tetrodotoxin; MEC, mecamylamine; PIR, pirenzepine).

We then examined the effects of GDP-β-S on the oscillatory activity of PPN neurons. We used an extracellular solution containing SB, TTX, MEC, and PIR. GDP-β-S was administered intracellularly in the pipette. Our results show that the GDP-β-S (1 mM) blocked oscillatory activity, on average, within 6.8 ± 0.7 min (*n* = 7) after gaining access to the intracellular compartment (Figure 6A, black record immediately upon patching but before GDP-β-S diffused into the cell, gray record 10 min after patching and GDP-β-S had diffused into the cell). The mean frequency of the oscillations for these cells was 27 ± 2 Hz before they were blocked by the GDP-β-S (Figure 6B power spectrum, compare black pre-GDP-β-S vs. gray post-GDP-β-S line). These results show that G-proteins directly affect voltage sensitive P/Q-type calcium channels responsible for the oscillatory activity in PPN neurons. In addition to blocking PPN oscillations with GDP-β-S, we measured the frequency of PPN oscillations by activating G-proteins with GTP-γ-S (0.4 mM). This agent is known to reversibly activate G-proteins (31, 32). We used an extracellular solution containing SB, TTX, MEC, PIR, and CAR. GTP-γ-S was administered intracellularly through the pipette (like GDP-β-S). We waited ∼10 min for the GTP-γ-S to diffuse into the cell before we induced oscillations using current ramps. The first ramp was considered as minute 0 and a single ramp was recorded every minute for up to 35 min (Figure 6C). The power spectrum of these records shows that GTP-γ-S did not further increase the frequency of oscillations (Figure 6D). Over the extended period of recording, a gradual decrease in the amplitude of oscillations was observed (Figure 6D). However, this was probably due to intracellular rundown of I_Ca_ under these protocols and not due to GTP-γ-S, since we have observed comparable results in our control cells without GTP-γ-S. Our results showed that the mean frequency of PPN oscillations in the presence of intracellular GTP-γ-S (38 ± 1 Hz; *n* = 7) was not significantly different from the frequencies reported under persistent activation of M2 cholinergic receptors (40 ± 1 Hz; *n* = 11) (Figure 1C) (One-way ANOVA; *df* = 17, *t* = 6.7, *p* = 0.6). We conclude that current ramp-induced PPN oscillations did not show an increase in frequency, even after G-proteins had been activated by GTP-γ-S. It is possible that these cells had reached their plateau of activation after the persistent or long duration application of CAR.

## Discussion

These results show that, (a) persistent activation of muscarinic M2 muscarinic receptors caused the increase in oscillatory frequency previously shown in PPN neurons (3), and (b) an intracellular mechanism involving voltage-dependent activation of G-proteins mediated this increase in oscillatory frequency. That is, G-protein activation apparently changed both the kinetics and inactivation of P/Q-type calcium channels, suggesting that G-protein pathways are necessary for maintaining gamma band frequency oscillations in the PPN. This is the first time such physiological characteristics have been described in PPN neurons. Moreover, we also describe a dual effect of CAR (30 μM) (acute vs. persistent) on PPN oscillations. The significance of these findings is amplified in light of the recent discovery of similar oscillatory mechanism in the Pf nucleus, through which PPN projections are relayed throughout the cortex. PPN neurons show increased firing during waking and paradoxical sleep (33), while lesions of the PPN reduced or eliminated paradoxical sleep (34). The accumulated evidence from our studies suggests that the PPN nucleus could play an important role in maintaining gamma band activity during waking and REM sleep.

Gamma band activity is characterized by high frequency oscillations ( >30 Hz), which are thought to represent the mechanism for binding patterns of activity among distant neuronal groups into conscious perception (35–39). Gamma band activity has been described in cortical (40, 41), hippocampal (42), and cerebellar neurons (43). In our previous study, we identified the ionic channels responsible for high frequency oscillations in PPN neurons (3). We demonstrated that mainly P/Q-type, and to a lesser extent, N-type calcium channels mediated the rising phase, and delayed rectifier-like potassium channels mediated the falling phase of current ramp-induced oscillations (3). Our results suggested that these calcium channels were located in dendritic compartments and, in order to be open, we had to depolarize the soma to around −20 mV or beyond. Using longer 30 s current ramps, we were able to steadily depolarize the membrane and detect oscillations at ∼−40 mV membrane potential at the cell body. Thus, ionic channels necessary for oscillations in the PPN can be recruited during long lasting gradual depolarization, even in the absence of APs (i.e., in the presence of TTX), which further confirmed that current ramps are a useful tool for *in vitro* studies of physiological characteristics of ionic channels in distal dendrites.

### Acute effect off CAR

Carbachol activates both types of cholinergic receptors: muscarinic (metabotropic), and nicotinic (ionotropic) (2, 11, 19, 27). The most abundant receptor type in the PPN is the M2 muscarinic receptor, which is G_i_-protein coupled. M2 receptors are known to hyperpolarize the cell membrane by increasing membrane conductance for potassium ions while reducing calcium currents (44). Activation of M1 receptors has been reported to decrease membrane conductance by blocking potassium currents (6). In our study, we demonstrated that acute application of CAR reversibly blocked PPN oscillations. We speculate that temporary membrane hyperpolarization, caused by M2 receptor activation, is a possible explanation for the acute effect of CAR on the oscillatory activity in PPN neurons. Furthermore, blockade of G-protein activation by GDP-β-S disrupted the generation of oscillations in the PPN, suggesting that normal turnover of G-proteins is required to generate and sustain such oscillations. In the dendrites of hippocampal neurons, CAR has been shown to modulate calcium entry and intracellular [Ca^2+^] by activating signaling pathways linked to muscarinic receptors (45). Here we show that acute CAR application can block P/Q-type calcium channel-mediated oscillations in the PPN through M2 muscarinic receptors, and assume that, on a functional basis, brief, or phasic cholinergic input to PPN cells leads to an inhibition of high frequency oscillations.

### Persistent effect of CAR

In this study, we showed that P/Q-type calcium channels are responsible for the generation of PPN high frequency oscillations under persistent exposure to CAR. A specific P/Q-type Ca^2+^ channel blocker abolished oscillatory activity in the PPN. Therefore, we conclude that CAR did not change the types of channels that mediate high frequency oscillations, only their properties. In our previous study, we showed that persistent exposure to CAR did not change the amplitude of PPN oscillations compared to non-CAR conditions (3). We speculate that the number of Ca^2+^/K^+^ channels involved in this oscillatory activity did not change after persistent application of CAR. However, the frequency of these oscillations did increase. This increase in frequency of oscillations indicates that CAR changed the kinetic properties of these channels. More specifically, we demonstrated that muscarinic and not nicotinic receptors are responsible for the increase in oscillatory frequency in PPN neurons. One of the main goals of this study was to identify which muscarinic receptors are responsible for such behavior. Our data showed that M2 (G_i_-protein coupled) receptors can effectively block calcium channel-mediated oscillations, and also increase the frequency of oscillations by activating intracellular mechanisms. This dual effect of the M2 muscarinic receptor has never been reported in the PPN nucleus. We assume that continuous cholinergic activation of the PPN through the M2 receptors plays a key role in maintaining high frequency activity among PPN neurons.

### G-proteins

Carbachol reduced calcium currents in PPN neurons as previously described in other cell types (17, 29, 30). Indeed, it has been demonstrated that membrane-delimited inhibition of voltage-dependent calcium channels is affected by G_βγ_ subunits of neurotransmitter activated G-proteins (12–16). In our hands, no oscillations were observed in the presence of GDP-β-S, suggesting that availability of G-proteins is key to generating high frequency oscillations in the PPN. Using a three-pulse protocol extensively used by other authors (14–16), we found that CAR reduced calcium current amplitude of the pre-pulse more than the post-pulse, suggesting a voltage-dependent G-protein mechanism. In addition, CAR slowed the kinetics of the I_Ca_ without affecting calcium current amplitude in I2/I1 ratios. The effect of persistent CAR on calcium currents was prevented after adding GDP-β-S, and modulated similar effects on calcium currents only mediated by P/Q-type calcium channels. It is important to emphasize that calcium current density values after CAR were similar in the presence or absence of ω-CgTX-GVIA. This novel result might explain why the persistent CAR effect did not induce a total blockade of oscillations. Partial blockade of P/Q-type channels and slower activation/deactivation kinetics than in control conditions would provide a mechanism for inducing faster frequencies of oscillations in the presence of CAR. In addition, G-protein modulation of potassium channels might also provide the faster membrane potential repolarization necessary to sustain higher frequencies of oscillations. Further experiments are needed to further support this hypothesis.

### Limitations

There are some limitations in this study that need to be recognized. First, we conducted our experiments on neonatal neurons in PPN nucleus (9–12 days). This age corresponds to the largest developmental decrease in REM sleep in rats. Although these data can give us an idea about the physiological properties of these neurons early in the development of the animal, we do not know how CAR will affect the oscillatory activity of fully developed adult neurons. Extensive myelination of neurons during development ( >14 days) and in adulthood, especially in the brainstem, renders patch clamping of these neurons extremely difficult. Therefore, our conclusions regarding the significance of cholinergic modulation of RAS oscillatory activity needs to be considered with caution until these data are confirmed in adult animals. Another limitation is that our study was conducted on brain slices, which do not exhibit sleep-wake cycles, or perform other physiological functions, like movement. Therefore, it is difficult to link single cell activity of these cells to their functions *in vivo*. Future experiments, first in whole populations *in vitro* and then *in vivo* could give us a better understanding of mechanism behind gamma band activity in the RAS. Ultimately, more experiments are needed to confirm our findings, however, the results described here provide novel insights into the electrophysiological properties of neonatal PPN neurons, and a role for the cholinergic system in the propagation of high frequency oscillations between the nuclei of the RAS.

### Functional and clinical implications

The implications of these results are significant. The marked differences between the manifestations of oscillations in PPN cells during acute vs. persistent exposure to CAR may be related to the presence of short duration/phasic vs. long duration/tonic cholinergic input. That is, short duration bouts of cholinergic input to PPN neurons may tend to block their capacity to oscillate at high frequencies, however, under persistent, long duration, or tonic cholinergic influence, PPN neurons could oscillate at higher frequencies, especially in the gamma range. It is not clear if phasic cholinergic tone is more characteristic of patterns observed during REM sleep, in which PPN cells burst more, or if tonic cholinergic tone is more characteristic of waking, when PPN cells tend to fire more tonically. The intracellular mechanism behind this effect appears to be related to G-proteins. The fact that inactivation of G-proteins with GDP-β-S blocked oscillations, but stimulation of G-proteins with GTP-γ-S had no effect, suggests that G-protein binding can plateau, beyond which no blockade of oscillations is possible. We hypothesize that, under short duration, acute, or phasic exposure to cholinergic input, G-proteins can still bind calcium channels, slowing their activation. This would reduce or prevent the induction of high frequency oscillations through P/Q-type calcium channels. However, under long duration, tonic cholinergic input, G-proteins may be bound to the persistent input, maximizing their utilization, thereby freeing calcium channels to become activated at their membrane potential threshold, leading to sustained gamma frequency oscillatory activity. Much additional information is needed to support this speculation, which must remain a working hypothesis.

Clinically, ADHD patients manifest an increase in slow wave (delta, theta), and a decrease in higher frequency (alpha, beta, gamma) activity (46). Reduced gamma band activity also has been reported in bipolar disorder (47). Aberrant gamma band activity and coherence during cognitive tasks or attentional load have been reported in schizophrenics (48). Several human studies demonstrated frequency-specific deficits in the coherence and maintenance of gamma oscillations in patients with schizophrenia (49). These disorders manifest symptoms such as hyperarousal, increased REM sleep drive, and decreased slow wave sleep, among others, suggesting involvement of the PPN (1). In developing novel therapies for these disorders, a potential target is the regulation of voltage-dependent P/Q-type calcium channels leading to high frequency activity through a permissive mechanism of G-protein occupation by continuous cholinergic input. Dysregulation at any point in this complex mechanism could account for some or all of the sleep-wake symptomatology in some or all of these disorders, highlighting the potential importance of the findings described.

In summary, the effect of CAR on PPN oscillatory activity is mediated by a G-protein voltage-dependent partial reduction of P/Q-type calcium current amplitude. These novel results support the idea that both P/Q-type channels and G-protein pathways are needed in order to sustain high frequency oscillations in PPN neurons. Moreover, these results suggest that persistent cholinergic input creates an active mechanism that allows the fine tuning of P/Q-type calcium channel-mediated oscillations.

## Conflict of Interest Statement

The authors declare that the research was conducted in the absence of any commercial or financial relationships that could be construed as a potential conflict of interest.
